# Mechanisms of cell entry by human papillomaviruses: an overview

**DOI:** 10.1186/1743-422X-7-11

**Published:** 2010-01-20

**Authors:** Caroline AJ Horvath, Gaëlle AV Boulet, Virginie M Renoux, Philippe O Delvenne, John-Paul J Bogers

**Affiliations:** 1 Applied Molecular Biology Research (AMBIOR) group, Laboratory for Cell Biology and Histology, University of Antwerp, Antwerp, Belgium; 2 Laboratory for Clinical Pathology (Labo Lokeren, campus RIATOL), Amerikalei 62-64, B-2000 Antwerp, Belgium; 3 Department of Pathology, University of Liège, Liège, Belgium

## Abstract

As the primary etiological agents of cervical cancer, human papillomaviruses (HPVs) must deliver their genetic material into the nucleus of the target cell. The viral capsid has evolved to fulfil various roles that are critical to establish viral infection. The particle interacts with the cell surface via interaction of the major capsid protein, L1, with heparan sulfate proteoglycans. Moreover, accumulating evidence suggests the involvement of a secondary receptor and a possible role for the minor capsid protein, L2, in cell surface interactions.

The entry of HPV *in vitro *is initiated by binding to a cell surface receptor in contrast to the *in vivo *situation where the basement membrane has recently been identified as the primary site of virus binding. Binding of HPV triggers conformational changes, which affect both capsid proteins L1 and L2, and such changes are a prerequisite for interaction with the elusive uptake receptor. Most HPV types that have been examined, appear to enter the cell via a clathrin-dependent endocytic mechanism, although many data are inconclusive and inconsistent. Furthermore, the productive entry of HPV is a process that occurs slowly and asynchronously and it is characterised by an unusually extended residence on the cell surface.

Despite the significant advances and the emergence of a general picture of the infectious HPV entry pathway, many details remain to be clarified. The impressive technological progress in HPV virion analysis achieved over the past decade, in addition to the improvements in general methodologies for studying viral infections, provide reasons to be optimistic about further advancement of this field.

This mini review is intended to provide a concise overview of the literature in HPV virion/host cell interactions and the consequences for endocytosis.

## Introduction

Human papillomaviruses (HPVs) are small, non-enveloped double-stranded DNA viruses that belong to the *Papovaviridae *family [1,2]. Scientific evidence accumulated from virological, molecular, clinical and epidemiological studies has identified HPV as the primary etiological agent in cervical cancer [1,3,4].

Like other viruses, HPVs are obligatory intracellular parasites and must deliver their genome and accessory proteins into host cells and subsequently make use of the biosynthetic cellular machinery for viral replication [5,6]. The journey of a HPV particle from the cell surface to the cytosol and nucleus consists of a series of consecutive steps that move it closer to its site of replication. The viral capsid plays a key role in the establishment of the viral infection [5,7].

By analyzing virus-cell interactions and uptake mechanisms, much can be learned about the biology of HPV replication and entry pathways, providing a means to discover unique ways for exploiting or interfering with the viral pathogenesis [5,6].

The HPV genome is surrounded by an icosahedral capsid (T = 7) of 55 nm in diameter composed by two structural proteins, the major protein L1 and the minor capsid protein L2 [8]. The L1 proteins are highly conserved and form 72 five-fold capsomers. The L2 protein is an internally located multifunctional protein with roles in genome encapsidation [9-11], L1 interaction and capsid stabilization [12,13], endosomal escape of virions [14,15] and nuclear transport of the HPV genome [15,16]. Viral capsids have evolved to fulfil numerous roles that are critical to the establishment of viral infection. For non-enveloped viruses, such as HPVs, the proteinaceous coat encases and protects the viral nucleic acid and provides the initial interaction site of the viral particle with the host cell. After receptor engagement the virus is internalized and its coat is disassembled to allow the encapsidated genome access to the cellular transcription and replication machinery [17].

Infectious HPV particles entry appears to occur specifically in the basal keratinocytes of the mucosal epithelium subsequent to the binding of virions to the basement membrane of the disrupted epithelium [9,18]. Since HPV replication and assembly requires infected basal keratinocytes to undergo the stepwise differentiation program of the epithelium [19,20], HPV propagation in cell culture is a major challenge. The production of infectious virus particles or virions was impossible until the development of organotypic raft cultures based on keratinocytes harbouring HPV genomes. However, these methods are technically demanding, time-consuming and they only produce relatively limited amounts of virions. These limitations were partially overcome by the use of DNA-free virus-like particles (VLPs) and by pseudovirions (PsVs) harbouring reporter plasmids, which were generated using heterologous expression systems [21,22]. These VLPs and PsVs have very similar structural and immunological characteristics to native HPV virions [8]

Condon optimization of capsid genes yielded high-level expression of capsid proteins and the development of packaging cell lines harboring high copy numbers of packaging plasmids finally allowed the large-scale production of PsVs and, subsequently, quasivirions (QVs), which are "quasi" identical to the authentic HPV virions [8,21-23]. This has prompted many researchers to study the HPV-host cell interaction by using VLPs, PsVs or QVs.

### HPV-host cell interactions

#### Cell surface binding: receptors

**Figure 1:**
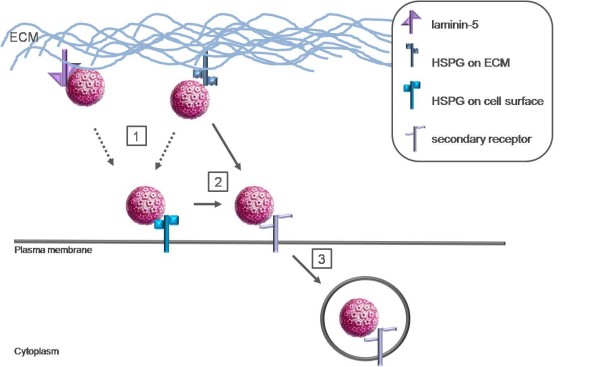
**Putative model of interaction of HPV capsids with the ECM and cell surface**. **1) **HSPG, a widely expressed and evolutionary conserved cell surface receptor, is suggested as the initial attachment receptor for HPVs and is frequently found in the ECM and on the surface of most cells. HPV capsids have also been shown to bind to ECM-resident laminin-5 although this interaction seems to be of lesser importance for a productive infection. **2) **Accumulating evidence suggests that a secondary receptor is involved in the infectious entry of HPV subsequent to HSPG interaction. The capsids are transferred to the putative secondary receptor on the cell surface. Whether transfer from primary ECM binding sites to primary cell surface binding sites occurs has not been directly investigated (dotted arrows). Capsid interaction with HSPG results in a conformational change that, in turn, results in the exposure of a furin cleavage site. Following this proteolytic cleavage, an additional conformational change exposes the binding site for the secondary cell surface receptor or lowers the affinity for the primary receptor which results in the hand-off to the second receptor, which then triggers endocytosis **3)**.

Host cell entry of HPV is initiated by binding of the virus particle to cell surface receptors (Figure 1). It has been suggested that virions bind initially to the basement membrane prior to transfer to the basal keratinocyte cell surface [18]. It is important to note that the entry of HPV *in vitro *is initiated by binding to a cell surface receptor in contrast to the *in vivo *situation where the basement membrane has recently been identified as the primary site of virus binding [18,21].

Early work investigating the cell surface receptors found that HPVs bind to a widely expressed and evolutionary conserved cell surface receptor and that the interaction depends primarily on L1 [24-27]. Glycosaminoglycans (GAGs), especially heparan sulfate, were suggested as initial attachment receptors for HPV VLPs [28-31]. Heparan sulfate proteoglycans (HSPG) are frequently found in the extracellular matrix (ECM) and on the surface of most cells. They are involved in several biological functions and because of their location they are appropriate molecules for viral infection [32,33]. Heparan sulfate is often found on two membrane-bound proteoglycans, syndecans and glypicans [34]. Glypicans are predominantly expressed in the central nervous system, whereas syndecans are the predominant HSPG in epithelial cells, the target cells of HPV. Especially syndecan-1 may serve as the primary attachment receptor *in vivo *due to its high expression level in the appropriate target cells and upregulation during wound healing [27,35]. Furthermore, other candidate receptors for HPV have been suggested, such as laminin-5. Several *in vitro *studies have shown that HPV can also bind to a receptor in the ECM, identified as laminin-5 which is able to mediate binding to the ECM [36-38]. However, laminin-5 interaction seems to be of lesser importance for a productive infection and even though the affinity to laminin-5 is higher than to heparan sulfate, infectious transfer from the ECM seems to require heparan sulfate binding [27,37,38].

The classical notion of a virus binding to a single receptor to enter cells through a single defined uptake mechanism is quickly being overtaken by a more complex picture. New findings, such as a specific co-receptor and virus attachment to multiple receptors, have raised the question that viruses known to bind to a non-specific receptor may turn out to also have a more specific co-receptor [39].

Like HPVs, mammalian herpesviruses adsorb strongly to proteoglycans, especially HSPGs. For the herpes simplex virus (HSV) this high affinity attachment step enhances infectivity, although it does not appear to be an absolute requirement for the virus to infect the cell. HSPG is preferred and is considered to be a binding receptor, as opposed to an entry receptor. It is obvious that for cell penetration, HSV usually interacts with co-receptors that are distinct from the proteoglycans attachment receptor [7,40].

Accumulating evidence suggests that a secondary receptor or co-receptor is also involved in the infectious internalization of HPV subsequent to interaction with HSPG [38,41]. It appears that HSPG functions as more than a simple attachment factor in HPV infection in that this interaction promotes essential conformational changes in the viral capsid, but HSPGs are clearly not the cell surface receptors that mediate virion internalization or later events in infection [41].

The cell adhesion receptor α6-integrin, which is involved in cell to cell interactions, has been suggested as secondary receptor even though its involvement in HPV infection is rather controversial [29,35,37,42-44]. Given the close association of proteoglycans and integrins as matrix components, it is possible that the experimental association of α6-integrin with HPV binding and entry is a secondary effect due to its interaction with HSPGs [7].

Several studies suggest a role for L2 in facilitating infection via interaction with a secondary receptor(s) [45-48]. Although cell surface interactions predominantly depend on the major capsid protein L1, it seems likely that the secondary cell surface receptor is L1-specific, although, it is possible that L2 may contribute to surface interactions [21].

These observations could indicate that the cell surface binding is indeed mediated by more than one receptor. A reasonable hypothesis is that a productive infection would require an initial low specificity binding mediated by L1, followed by the interaction of a more specific protein component with L2 [7]. A specific region in the L2 protein was proposed to interact with a cell surface molecule after attachment of the virus to a primary receptor. This interpretation suggests a post-attachment conformational change at the cell surface to unmask this specific domain in L2, a process that many other viruses use to trigger downstream events such as secondary receptor interactions [27,48].

Initial attachment to HSPG moieties functions primarily to facilitate the critical step of L2 proteolytic cleavage which is essential for successful infection [41]. The minor capsid protein L2 is cleaved by furin on the cell surface at a consensus cleavage site that is conserved among all papillomaviruses [17]. These sequences are inaccessible at the surface of mature virions in solution in order to prevent host antibody response to the conserved epitopes [27]. As mentioned above, capsid interaction with HSPG results in a conformational change which results in the exposure of the furin cleavage region. After cleavage, an additional conformational change may expose the binding site for the secondary cell receptor, or it lowers the affinity for the primary receptor, which results in the hand-off to a secondary receptor [27,41,49].

Taken together, capsid interaction with HSPG induces conformational changes that result in the exposure of the L2 amino terminus. Exposure of this L2 N-terminus allows access to highly conserved consensus furin convertase recognition site and subsequent furin cleavage which is essential for successful infection. Moreover, the L2 N-terminus is essential for the L2 protein to adopt a correct conformation within the assembled capsid. Correct folding may also require the formation of a disulfide bond between HPV16 L2 cysteine residues Cys22 and Cys28, which was recently identified. Mutation of the contributing cysteine residues rendered mutant virions non-infectious [15,21,50,51].

Even if keratinocytes are the main targets of HPV and only entry in these cells has been shown to result in a productive infection, HPV-VLP are also able to enter other cellular types such as dendritic cells (DC) or Langerhans cells (LC). Interactions between these antigen presenting cells (APCs) and HPV are likely to be important for the establishment of the immune response after a prophylactic vaccination or a natural infection. Bousarghin *et al*. showed that these APCs differentially interact with HPV16 VLPs. Although DC and LC are able to bind and internalize HPV16 VLPs, there are differences in VLP binding to DC and LC. DC use heparan sulfates to bind HPV16 VLPs in contrast to LC on which heparin does not have any inhibitory effects [52]. Various studies showed that VLPs co-localize with langerin in LC [52,53]. Although still controversial, the investigation on the immunogenicity of VLPs supports a key contribution for the low-affinity Fcγ receptors, expressed on DC, as an important molecule in a HPV-VLP receptor complex [54,55].

#### Internalization

After binding to cell surface receptors HPV must be internalized into the cell to establish an infection. To date, the dynamics of HPV interaction with the cell surface during the initial stages of infection are not completely understood and the entry mechanisms and the molecules involved are contradictory and still a subject of scientific debate (table 1).

**Table 1:** Overview HPV internalization studies

HPV type	Methods	Pathway	Reference
HPV16	siRNA-mediated down regulation of clathrin heavy chain/caveolin-1/dynamin/tetraspanins dominant negative mutants of EPS15/caveolin-1/dynamin biochemical inhibitors caveolae-deficient cells	clathrin- and caveolae-independent dynamin-independent lipid raft independent involvement of tetraspanins	[58]
HPV16 HPV31	biochemical inhibitors	clathrin-dependent	[66]
HPV16 HPV31	dominant negative mutant of EPS15/caveolin-1/dynamin-2 biochemical inhibitors co-localization studies of HPV16 and HPV31 association study of HPV31 with detergent resistant microdomains	HPV16 clathrin-dependent HPV31 caveolae-dependent	[65]
HPV16	co-localization with BPV-L1 VLPs	clathrin-dependent	[63]
HPV16 HPV31 HPV58	biochemical inhibitors microscopic analysis	HPV16/58 clathrin-dependent HPV31 caveolae-dependent	[64]
HPV33	biochemical inhibitors	non-caveolae dependent HPV33 uptake	[59]

The conflicting data could be due to the "maturity" state of the VLPs and PsVs used. HPV capsids extracted from replicating cultured cells can exist in two forms. "Immature" capsids are larger, less regular and less protease resistant than "mature" capsids indicating a substantial change in conformation during the maturation process [56]. Therefore, it is likely that the omission of a maturation step could result in assay variability due to particle heterogeneity [7]. Moreover, HPVs exhibit promiscuous cell association while only completing their life cycle in differentiating squamous epithelium [57]. Therefore, while the early events of infection may be similar in permissive and non-permissive cell types, there is a restriction of viral replicative functions and virion production that is determined by factors tied to the keratinocyte differentiation program [7].

Productive entry of HPV involves internalization by endocytosis, a process that for HPV occurs slowly and asynchronously over a period of several hours, except for some non-epithelial cells [8,52,58]. Multiple studies have shown an unusually extended residence on the cell surface for HPVs [7,29,59,60]. Most ligands, including the majority of viruses, are internalized rapidly, within minutes after the initial receptor encounter and engagement. The reason for the delayed kinetics for HPVs is unknown, although it is noteworthy that syndecans have been reported to have a slow rate of internalization after ligand binding [61]. Alternatively, the conformational changes or the transfer to a secondary receptor that is sparsely arrayed or exhibits particular requirements for endocytosis are a possible explanation for the slow kinetics [8,27,58]. Moreover, *in vitro *experiments showed that cell surface dynamics of HPV indicated a transport mechanism along actin rich cell protrusions to access the endocytic machinery and thus enhance infectious entry. This transport was facilitated by binding to receptors that were likely to interact with actin filaments to mediate the transport towards the cell body by retrograde flow. This requirement may contribute to the prolonged residence on the cell surface and the impeded kinetics [8].

Several endocytic pathways have been described and clathrin- and caveolae-mediated are two main pathways used by non-enveloped viruses to infect cells [5,62]. A possible approach to distinguish between the clathrin-dependent and caveolar pathways is the analysis of biochemical inhibition of ligand uptake, although non-specific effects must be considered. The development of molecular inhibitors in the form of dominant-negative molecules has surpassed the use of biochemical inhibitors in terms of decreasing these non-specific effects. Selinka *et al*. examined a set of biochemical inhibitors for effects on HPV33 PsV infection and found a dependence upon an intact actin cytoskeleton and microtubules. Day *et al*. investigated the uptake of bovine papillomaviruses (BPVs) through biochemical inhibitor analysis and co-localization studies with established markers of endocytic compartments. Both studies could not demonstrate the involvement of caveolar endocytosis and concluded that uptake of these viruses occurs via a clathrin-dependent pathway [59,63]. However, a study utilizing PsVs, generated by mixing VLPs with naked DNA, unexpectedly found that HPV31 was sensitive to caveolar inhibition. In contrast, the entry of HPV16, which phylogenetically, is closely related to HPV31, and HPV58 was found to be blocked by inhibitors of clathrin-mediated uptake [64]. The data on the entry of HPV31 was confirmed by Smith *et al*. who described a caveolar uptake of HPV31 virions in keratinocytes [65]. However, another study found that biochemical inhibition of clathrin-dependent uptake did prevent HPV31 infection [66]. HPV31 appears to interact with HSPG similarly to HPV16 for *in vivo *infection. Possibly HPV31 interacts differently with or has a unique co-receptor that shunts it into a different internalization pathway [67].

Most studies investigating the uptake of HPV16 concluded the involvement of clathrin-dependent endocytosis [63-66]. In contrast to these studies, Spoden *et al*. observed clathrin- and caveolae-independent internalization of HPV16 PsVs. Entry occurred by a mechanism that was resistant to combined siRNA-mediated down regulation of caveolin-1 and clathrin heavy chain as well as being resistant to over-expression of dominant negative mutants of caveolin-1 and eps-15, which plays a role in clathrin coated vesicle formation [58]. The authors suggested the involvement of tetraspanin-enriched microdomains that serve as a platform for uptake by an uncharacterized internalization mechanism. None of the conducted studies demonstrated an effect of caveolar disruption on HPV16 infection.

Initiation and progression of HPV-associated cervical cancer have been shown to be related to functional alterations of LC within the cervical epithelium. Because of their role in initiating an antiviral immune response, DC and LC represent an ideal target for immune evasion by viruses. The study of the interactions between HPV16 VLPs and DC or LC showed that the entry of virus particles is different as suggested by Fausch *et al*. and Yan *et al*. Fausch *et al*. showed that DC use a clathrin-mediated endocytosis whereas LC use a different pathway which is not associated with clathrin or caveolae [68]. Yan *et al*. show that LC uptake of HPV6 L1 was blocked by filipin pretreatment confirming a role for caveolin-mediated uptake of VLPs by LC [53]. Another study, however, showed that virus particles use the same clathrin-dependent endocytic pathway to enter DC and LC [52].

## Conclusions

The most likely scenario for HPV entry includes cell surface binding of virions mediated via HSPGs. This primary attachment is dependent only on L1 and does not require L2. A long delay in internalization is accompanied by changes in the mode of binding and possible transfer to a secondary receptor. Although there is as yet no evidence, it is suggestive that L2 is involved in this early process. The most likely scenario is that the conformational changes in L2 that occur on the cell surface are necessary to expose a secondary binding site.

HPVs are generally internalized via a clathrin-dependent endocytic mechanism, which is initially dependent on actin. Some HPV types may use alternative uptake pathways to enter cells, such as a caveolae-dependent route or the involvement of tetraspanin-enriched domains as a platform for viral uptake.

Despite the significant advances and the emergence of a general picture of the infectious entry pathway of HPV, many details remain to be clarified. The studies necessary to elucidate the ambiguous features concerning HPV binding and entry will be technically challenging. However, the remarkable technological advances in HPV virion analysis achieved over the last decade, in addition to the improvements in general methodologies for studying viral infections, provide reasons to be optimistic about further advancement in the field of HPV binding and entry. However, even with these advances ambiguity and a reason for caution still remains. The plasticity of many cellular pathways means that viral entry may be impacted by an indirect mechanism rather than by direct inhibition. Moreover, it is possible that HPVs make use of multiple internalization pathways. The next advancements in the study of HPV entry are the developments in real-time single molecule imaging of viral infections, which provide an extra level of sophistication and allow viewing entry and subsequent trafficking of HPV into live cells with exquisite clarity.

## List of abbreviations

HPV: Human papillomavirus; L1: Late protein 1; L2: Late protein 2; DNA: Deoxyribonucleic acid; VLP: Virus-like particle; PsV: Pseudovirion; QV: Quasivirion; GAG: Glycosaminoglycan; ECM: Extracellular matrix; HSPG: Heparan sulfate proteoglycan; HSV: Herpes Simplex virus; Cys: Cysteine; DC: Dendritic cells; LC: Langerhans cells; APC: Antigen-presenting cell; BPV: Bovine papillomavirus; siRNA: small interfering RNA.

## Competing interests

The authors declare that they have no competing interests.

## Authors' contributions

CH conceived of the study, and participated in its design, coordination and writing.

GB has been involved in revising the manuscript critically for important intellectual content.

PD has been involved in revising the manuscript critically for important intellectual content.

VR has been involved in revising the manuscript critically for important intellectual content.

JB has been involved in revising the manuscript critically for important intellectual content and has given final approval of the version to be published.

All authors read and approved the final manuscript.
